# Effect of continuity of care based on IKAP theory on caregiver burden and symptom management in patients with chronic obstructive pulmonary disease

**DOI:** 10.3389/fmed.2026.1858995

**Published:** 2026-06-29

**Authors:** Qiaoling Zhang, Huafei Yan, Chenfang Li

**Affiliations:** 1 Department of Respiratory and Critical Care Medicine, The First People’s Hospital of WuYi County, Zhejiang, China; 2 Department of Nursing, The First People’s Hospital of WuYi County, Zhejiang, China; 3 Department of Emergency, The First People’s Hospital of WuYi County, Zhejiang, China

**Keywords:** caregiver burden, chronic obstructive pulmonary disease, continuity care, IKAP theory, symptom management

## Abstract

**Objective:**

To examine the effect of continuity care guided by Information–Knowledge–Attitude–Practice (IKAP) theory on symptom management in patients with chronic obstructive pulmonary disease (COPD) and on caregiver burden.

**Methods:**

A retrospective controlled study was conducted. A total of 300 COPD patients discharged from one hospital between April 2024 and June 2025 were included. Patients were assigned to routine care (*n* = 150) or IKAP-based continuity care (*n* = 150). The intervention group received structured measures, including information collection, knowledge delivery, attitude reinforcement, individualized exercise prescriptions, and caregiver training, maintained for 6 months. Outcomes included caregiver burden (Zarit Burden Interview-22, ZBI-22), COPD-related health status (COPD Assessment Test, CAT), disease-specific quality of life (St. George’s Respiratory Questionnaire, SGRQ), disease knowledge level, medication adherence (Adherence to Refills and Medications Scale, ARMS), and self-care ability (Exercise of Self-Care Agency, ESCA). Assessments were conducted at discharge (T0), 1 month (T1), 3 months (T2), and 6 months (T3).

**Results:**

The intervention group had higher rates of nursing contact, continuity achievement rate, completion of knowledge delivery, adherence to exercise prescription, and caregiver training compared with the control group (*P* < 0.001). In the intervention group, ZBI-22 and CAT scores were significantly lower than in the control group at T1–T3 (all *P* < 0.001) and showed a continuous decline. SGRQ dimensions and total scores improved progressively, with a total score of 43.4 ± 10.9 at T3, which was lower than 60.3 ± 11.7 in the control group (*P* < 0.001). The proportion of patients with excellent knowledge increased from < 20% at T0 to 58.0% at T3 in the intervention group, higher than in the control group (*P* < 0.001). High adherence increased from less than 30% at baseline to 64.0% at T3 in the intervention group, while no clear improvement was observed in the control group. ESCA scores reached 122.1 ± 10.2 at T3 in the intervention group, compared with 95.0 ± 8.1 in the control group (*P* < 0.001).

**Conclusion:**

In this 6-month observation, IKAP-based continuity care was associated with reduced caregiver burden and improved knowledge. For COPD patients, it was linked to better symptoms, quality of life, knowledge, medication adherence, and self-care ability. This model offers preliminary evidence for continuity care in chronic disease management.

## 1 Introduction

Chronic obstructive pulmonary disease (COPD) is one of the most burdensome chronic respiratory diseases worldwide. Its prevalence and mortality continue to increase, and it is expected to rank among the top four causes of death by 2030 (1, 2). Epidemiological surveys indicate that about 300 million people worldwide live with COPD (3). It is characterized by irreversible airflow limitation, chronic inflammation, and frequent acute exacerbations. These conditions not only reduce exercise tolerance, cause dyspnea, and limit daily function but also result in long-term declines in quality of life and increased healthcare costs (4). In China, COPD also shows high prevalence and disability rates, which place great pressure on the public health system (5, 6). Beyond the burden experienced by patients themselves, the long-term management of COPD relies heavily on family caregiving. Caregivers often face psychological stress, sleep disturbances, and physical fatigue while undertaking tasks such as daily care, medication management, and responding to acute exacerbations. These problems may reduce the quality of care and increase socioeconomic burden (7, 8). Previous studies have shown that discharge instructions or one-time health education have limited long-term effects. Symptom control and adherence in patients improve only slightly, and caregiver support is also insufficient (9, 10). Therefore, maintaining continuity of care after discharge while addressing both patient and caregiver needs has become a major clinical challenge.

In recent years, continuity care has gained attention for extending nursing services into communities and households. Studies have suggested that continuity care involving remote follow-up, health education, and exercise prescriptions may improve quality of life and functional status to some extent (11). However, the outcomes vary widely. Some interventions lack a theoretical framework, resulting in fragmented content and inconsistent implementation, which makes it difficult to establish a systematic and reproducible pathway. The Information–Knowledge–Attitude–Practice (IKAP) theory emphasizes the gradual transformation of health behavior through information collection, knowledge transfer, attitude reinforcement, and practice implementation (12). This theory has already shown positive effects in the management of chronic diseases such as diabetes, cardiovascular conditions, and postoperative rehabilitation after prostate surgery (13). Yet, evidence of its application in COPD nursing remains scarce. Most existing studies focus on single outcome indicators, with few addressing both patient symptom management and caregiver burden. Comprehensive care guided by a theoretical framework is especially lacking (14).

The aim was to provide evidence for continuity care in COPD and to offer references for innovations in chronic disease management and family–patient collaborative practice.

## 2 Materials and methods

### 2.1 Participants

**FIGURE 1:**
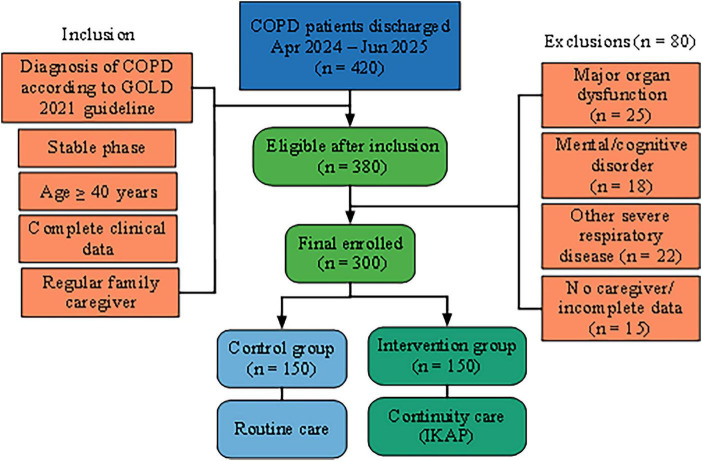
Flowchart of case screening.

This study adopted a retrospective controlled design. A total of 300 patients with COPD who were discharged from the Department of Respiratory and Critical Care Medicine of Wuyi County First People’s Hospital between April 2024 and June 2025 were included. Differences between routine nursing care and the continuous nursing model based on IKAP theory were compared in terms of caregiver burden, patient symptoms, health-related quality of life, medication adherence, and self-care ability. The study was approved by the Medical Ethics Committee of the hospital (Approval number: 20250908). Inclusion criteria: (1) diagnosis consistent with the Global Initiative for Chronic Obstructive Lung Disease (GOLD) guidelines (2021 revision), and patients in a stable stage (15); (2) age ≥ 40 years; (3) complete clinical records with ability to complete scale assessments; (4) availability of a fixed family caregiver after discharge. A fixed family caregiver was defined as a direct relative or long-term cohabiting family member who continuously provided daily care, including medication assistance, symptom observation, and rehabilitation support, with basic communication ability. Exclusion criteria: (1) severe dysfunction of heart, brain, liver, kidney, or other major organs; (2) psychiatric illness or cognitive impairment, assessed by the Mini-Mental State Examination (16), with a score of < 24 indicating cognitive impairment that limited completion of study-related assessments; (3) other severe respiratory diseases, such as lung cancer or bronchiectasis; (4) lack of fixed caregiver or incomplete follow-up data. Grouping was based on the nursing model recorded in the electronic medical records and the hospital nursing management platform. The control group (*n* = 150) included patients who received routine nursing care, whereas the intervention group (*n* = 150) included patients whose records showed that they had received the continuous nursing program based on IKAP theory. Patients in both groups were enrolled during the same study period (April 2024 to June 2025). Group allocation was determined according to the nursing model documented in the nursing records rather than by different time periods. Patients with incomplete clinical records or inability to complete follow-up assessments were excluded during the screening process. Consequently, all 300 participants included in the final analysis had complete baseline and follow-up data available for evaluation. The patient selection process is shown in Figure 1.

### 2.2 Methods

As this was a retrospective study, no new interventions were implemented during the study period. Instead, nursing records, electronic medical records, and nursing management platform data were reviewed to summarize and categorize different nursing models. The control group received routine nursing management after discharge. This involved one-time health education at discharge covering disease knowledge, medication use, and precautions, but no structured or continuous intervention was provided thereafter. The intervention group received continuity of care measures that had already been implemented by the nursing team in routine practice, in addition to standard nursing care. Based on previous records and platform data, the intervention pathway was summarized and structured in accordance with IKAP theory (Table 1).

**TABLE 1:** Continuity of care pathway guided by the IKAP theory.

Stage	Intervention objective	Intervention record time	Specific measures
Information	Collect individual care needs	Discharge baseline (T0) initial record; updated records at T1, T2, and T3	Establish patient files. Record basic information, lung function, rehabilitation goals, symptom characteristics, and complications. Dynamically update individualized management plans and retain records on the nursing platform.
Knowledge	Improve patients’ disease-related knowledge	Nursing records showed discharge baseline (T0), one to two times/month reminders; each point had follow-up records	Platform promoted basic knowledge of COPD. Provided exercise training, smoking cessation, alcohol reduction, diet, psychological support, and medication instructions. Delivered both online and offline education. Encouraged repeated learning and self-review.
Attitude	Enhance patients’ willingness for rehabilitation training	Discharge baseline (T0) initial record; nursing files updated every 4 weeks	Nursing records documented psychological support. Family members joined training sessions. Conducted health lectures and interactive group discussions to improve motivation.
Practice	Promote healthy behaviors	Weekly records at T1–T3	SMART goals were set. The platform and wearable devices recorded exercise performance. Videos were uploaded and reviewed by supervisors. The nursing team tailored individual prescriptions. Training included 2–3 daily breathing sessions, one Baduanjin exercise per day, 3–5 weekly aerobic sessions, and 2–3 weekly resistance training sessions. If adherence was below 75%, targeted reminders were given.
Caregiver Training	Strengthen family support	Discharge baseline (T0) initial training; reinforced at T1, T2, and T3	Nursing records documented caregiver training. Guidance included medication management, breathing exercises, diet, and recognition of adverse reactions. Improved caregivers’ ability to cooperate with patient rehabilitation.
Continuity	Ensure long-term management	Nursing records showed interventions at weeks 1, 2, 4, and 8; supplementary records for patients with severe disease	Patients were contacted multiple times within 0–8 weeks. Records documented intervention adherence. For those with adherence below 75% or poor disease control, the number of contacts was increased, and extra management measures were implemented.

The intervention was delivered by full-time nursing staff who had received unified departmental training. The training covered the standard operating procedures of each IKAP stage, platform operation methods, and communication skills. Consistency of intervention implementation was monitored through operation logs, check-in records, and regular supervision within the nursing management platform. When patient adherence fell below 75%, the system automatically generated a reminder for additional contact. The nursing team then provided individualized follow-up to maintain continuity and reproducibility of the intervention.

### 2.3 Outcome measures

To evaluate the impact of continuity of care guided by IKAP theory on symptom management in patients with COPD and on family caregiver burden, multidimensional scale data were obtained through electronic medical records and the hospital nursing management platform. Assessment points were standardized as follows: T0 (baseline at discharge, within ± 3 days), T1 (approximately 1 month, the nearest record within ± 7 days of T0), T2 (approximately 3 months, within ± 14 days), and T3 (approximately 6 months, within ± 21 days). Each scale recorded the original score, and change values were calculated as Δ = Tx - T0. The COPD disease knowledge questionnaire was independently developed by the research team based on the clinical guidelines for the diagnosis and management of COPD and routine continuous nursing practice. The questionnaire items were discussed and revised by respiratory physicians and nursing experts. Content validity was evaluated using the expert consultation method. The content validity index (CVI) was 0.81. Questionnaire reliability was assessed using Cronbach’s α coefficient, which was 0.83. Before the formal study, a pilot test was conducted in a small sample of patients to evaluate the comprehensibility and feasibility of the questionnaire. Appropriate wording modifications were made according to participant feedback. The specific scales, measurement methods, and purposes of application are shown in Table 2.

**TABLE 2:** Scales, measurement methods, and application purposes.

Scale name	Measurement content and method	Scoring method	Application purpose
ZBI-22 (27)	22-item self-report scale, completed by caregivers.	0–88 points. Higher scores indicated greater burden. The most recent valid record at each time point was collected.	Quantified caregiver burden. Core outcome indicator.
CAT (28)	8-item self-report questionnaire, completed by patients at each time point and recorded on the nursing platform.	0–40 points. Higher scores indicated more severe symptoms and greater impact.	Assessed the impact of COPD on health status and daily functional limitation.
SGRQ (29)	Included domains of symptoms, activity, and impact. Scores were automatically calculated by the system.	0–100 points. Higher scores indicated poorer health-related quality of life.	Evaluated health-related quality of life in COPD. Supplemented CAT results.
COPD Knowledge Questionnaire (Hospital Version)	The questionnaire consisted of 20 items in a multiple-choice format, covering disease recognition, risk factor identification, recognition of acute exacerbations, medication management, smoking cessation, pulmonary rehabilitation, breathing exercises, and self-management.	The percentage of correct responses was calculated as the number of correctly answered items divided by the total number of items × 100%. Based on the percentage score, knowledge levels were classified as excellent ( ≥ 80%), good (60–79%), and poor ( < 60%). The most recent valid score at each assessment time point was used, and the number and proportion of participants in each category were analyzed.	Assessed patient knowledge of disease management. Corresponded to the Information and Knowledge stage of IKAP theory.
ARMS (30)	Patients completed the 12-item ARMS questionnaire by self-report at each assessment time point to evaluate medication-taking and prescription refill behaviors. Data were obtained from nursing follow-up records or the clinical management platform.	Each item was scored on a 4-point Likert scale (1–4 points), yielding a total score ranging from 12 to 48, with higher scores indicating poorer medication adherence. Based on the total score, adherence was categorized into good adherence ( ≤ 20 points), moderate adherence (21–27 points), and poor adherence ( ≥ 28 points).	Evaluated medication adherence behavior. Corresponded to the Practice stage of IKAP theory. Served as an intermediate variable.
Exercise of Self-Care Agency Scale (31)	29-item self-care ability scale, scored using Likert method.	Converted into standardized dimension scores. Higher scores indicated stronger self-care ability.	Measured self-care ability. Corresponded to the Attitude and Practice stage of IKAP theory.

To further clarify the implementation status and continuity of the IKAP-based nursing intervention, a set of process indicators was established in addition to outcome measures. These included the number of nursing contacts, rate of continuity achievement, completion rate of knowledge dissemination, adherence to individualized exercise prescriptions, and completion rate of caregiver training (Table 3).

**TABLE 3:** Process indicators of continuity of care implementation.

Indicator name	Determination rules	Measurement unit and threshold	Data source and time point	Purpose
Nursing contact/platform interaction frequency	Total number of effective contacts between the nursing team and patients within 0–8 weeks. Effective contact was defined as a telephone or face-to-face interaction lasting ≥ 5 min, or a platform message reply, call, or video consultation. Each complete interaction was counted as one.	Times; recorded as mean ± standard deviation (mean ± SD)	Aggregated to the most recent record at each time point (T1, T2, T3); derived from nursing records and platform logs	Reflected intensity of nursing contact
Continuity achievement rate	Adherence rate = (actual completed interactions ÷ planned interactions) × 100%. When individual adherence was ≥ 75%, it was judged as “achieved.”	%; threshold = 75%	0–8 week cumulative statistics; platform schedules and completion records	Reflected execution of continuity
Knowledge push completion rate	Completion rate of knowledge modules × 100%. Completion was defined as viewing > 90% or confirming reading plus quiz completion. Verification accuracy ≥ 80% was required.	%	Most recent cumulative value at each time point; course and test records	Reflected implementation of Information and Knowledge stage
Individualized exercise prescription adherence	Adherence = (actual completed exercise days ÷ planned exercise days) × 100%. Completion was defined as ≥ 80% of planned duration per day.	%	Most recent 1-month cumulative record; exercise logs and device data	Reflected implementation of Practice stage
Caregiver training completion rate	Completion rate of caregiver training modules × 100%. Completion was defined as attending videos or lectures with > 90% attendance, and accuracy ≥ 80% in skill checklist or quizzes.	%	Most recent cumulative value at each time point; training attendance and test records	Reflected implementation of family support

### 2.4 Statistical analysis

All statistical analyses were performed using SPSS version 26.0. Continuous variables were tested for normality and are presented as mean ± standard deviation (mean ± SD). Baseline comparability between groups was assessed using independent-samples t-tests for continuous variables and chi-square tests for categorical variables. For continuous outcomes, including the Zarit Burden Interview-22 (ZBI-22), COPD Assessment Test (CAT), St. George’s Respiratory Questionnaire (SGRQ), and Exercise of Self-Care Agency Scale (ESCA), between-group differences at T1, T2, and T3 were evaluated using analysis of covariance (ANCOVA). To reduce the potential influence of confounding factors inherent to the retrospective study design, the models were adjusted for age, sex, GOLD stage, smoking status, hypertension, diabetes mellitus, baseline CAT score, and baseline SGRQ total score. Adjusted between-group differences, and 95% confidence intervals (95% CIs) were reported for each follow-up time point. For ordinal categorical outcomes, including knowledge level and medication adherence level, data are presented as frequencies and percentages, and between-group differences at each time point were compared using chi-square tests. All statistical tests were two-tailed, and a *P* < 0.05 was considered statistically significant.

## 3 Results

### 3.1 Baseline characteristics

To ensure the reliability of group comparisons, baseline analyses were first conducted on demographic characteristics, comorbidities, disease severity, and key scale scores at discharge. The analysis revealed no significant intergroup differences in demographic or clinical variables, including age, gender, body mass index (BMI), smoking status, presence of hypertension or diabetes, GOLD classification, duration of hospitalization, and discharge CAT and SGRQ scores (*P* > 0.05). These outcomes suggested that the initial clinical characteristics of the two groups were well balanced (see Table 4).

**TABLE 4:** Baseline characteristics of patients.

Characteristic	Control (*n* = 150)	Intervention (*n* = 150)	Statistic (*t*/χ ^2^)	*P*-value
Age (years, mean ± SD)	66.8 ± 8.7	67.2 ± 8.4	*t* = 0.41	0.682
Sex (male, n, %)	98 (65.3%)	101 (67.3%)	χ^2^ = 0.11	0.739
BMI (kg/m^2^)	23.4 ± 3.5	23.7 ± 3.6	*t* = 0.72	0.472
Smoking history (n, %)	84 (56.0%)	87 (58.0%)	χ^2^ = 0.09	0.763
Hypertension (n, %)	62 (41.3%)	65 (43.3%)	χ^2^ = 0.10	0.752
Diabetes mellitus (n, %)	36 (24.0%)	38 (25.3%)	χ^2^ = 0.05	0.817
GOLD stage (II/III/IV)	59/65/26	61/64/25	χ^2^= 0.04	0.981
Length of hospital stay (days, mean ± SD)	10.5 ± 3.2	10.8 ± 3.4	*t* = 0.74	0.461
CAT score at discharge (mean ± SD)	19.8 ± 6.4	20.1 ± 6.2	*t* = 0.39	0.698
SGRQ total score at discharge (mean ± SD)	54.6 ± 12.3	55.0 ± 11.9	*t* = 0.27	0.788

### 3.2 Implementation and continuity of nursing

On the basis of comparable baseline characteristics, further analysis was performed to compare the differences between the two groups in nursing implementation and continuity. The intervention group showed significantly higher nursing contact frequency, continuity achievement rate, knowledge push completion rate, exercise prescription adherence, and caregiver training completion compared with the control group. These differences were statistically significant (all *P* < 0.001) (Table 5).

**TABLE 5:** Implementation and continuity indicators of nursing intervention based on IKAP theory.

Indicator	Control group (*n* = 150)	Intervention group (*n* = 150)	Statistic (χ ^2^/*t*)	*P*-value
Nursing contact and platform interaction frequency (times, mean ± SD)	1.3 ± 0.7	6.8 ± 2.1	*t* = 28.54	< 0.001
Continuity achievement rate (adherence ≥ 75%, n, %)	12 (8.0%)	109 (72.7%)	χ^2^ = 151.26	< 0.001
Knowledge push completion rate (%)	15.3 ± 9.2	83.6 ± 12.4	*t* = 58.71	< 0.001
Exercise prescription adherence (%)	21.4 ± 10.5	78.2 ± 15.3	*t* = 41.22	< 0.001
Caregiver training completion rate (%)	10.7 ± 6.8	85.1 ± 11.6	*t* = 68.45	< 0.001

### 3.3 Caregiver burden and patient symptom management

There was no significant difference in ZBI-22 scores between the two groups at baseline (*P* > 0.05). From T1 onward, the intervention group showed significantly lower scores than the control group (*P* < 0.001). After adjustment for covariates, the between-group difference increased initially and then decreased over the follow-up period, reaching the largest magnitude at T2 (–8.9 points, 95% CI: –9.8, –8.0). Within-group analysis indicated that ZBI-22 scores in the intervention group decreased continuously at each subsequent time point compared with the previous assessment (all *P* < 0.05) (see Table 6).

**TABLE 6:** Comparison of ZBI-22 and CAT scores between the two groups (points, mean ± SD).

Time point	Control group (*n* = 150)	Intervention group (*n* = 150)	Adjusted between-group difference (95% CI)	*P*-value
ZBI-22				
T0	48.4 ± 3.5	49.5 ± 3.6	–	> 0.05
T1	47.8 ± 4.3	41.6 ± 3.8^a^	-6.0 (-6.9, -5.1)	< 0.001
T2	45.6 ± 3.2	36.4 ± 3.3^ab^	-8.9 (-9.8, -8.0)	< 0.001
T3	44.5 ± 3.7	39.5 ± 3.1^abc^	-4.8 (-5.6, -4.0)	< 0.001
CAT				
T0	19.9 ± 4.5	20.2 ± 4.1	–	> 0.05
T1	19.5 ± 4.3	16.4 ± 3.7^a^	-3.0 (-3.8, -2.2)	< 0.001
T2	18.5 ± 4.5	14.2 ± 4.2^ab^	-4.1 (-5.0, -3.2)	< 0.001
T3	18.2 ± 4.2	12.7 ± 3.8^abc^	-5.3 (-6.2, -4.4)	< 0.001

*^a^P* < 0.05 versus T0 within the same group.

*^b^P* < 0.05 versus T1 within the same group;

*^c^P* < 0.05 versus T2 within the same group. Adjusted between-group difference = intervention group - control group, adjusted for age, sex, GOLD classification, smoking status, comorbidities, baseline CAT score, and baseline SGRQ score.

No significant difference in CAT scores was observed between the two groups at baseline (*P* > 0.05). From T1 onward, CAT scores were significantly lower in the intervention group than in the control group (*P* < 0.001). The adjusted between-group difference increased progressively during follow-up and reached its maximum at T3 (–5.3 points, 95% CI: –6.2, –4.4). Within-group comparisons showed a continuous reduction in CAT scores in the intervention group across all follow-up assessments relative to the preceding time point (all *P* < 0.05) (see Table 6).

### 3.4 Health-related quality of life

No significant differences were observed between groups in any SGRQ domain or total score at baseline (all *P* > 0.05). From T1 onwards, all domain scores and total scores were significantly lower in the intervention group than in the control group (all *P* < 0.001), with between-group differences widening progressively over time. At T3, the covariate-adjusted between-group difference in total score was –16.3 points (95% CI: –18.8, –13.8), with the activity domain showing the largest difference (–18.2 points, 95% CI: –21.0, –15.4). Within-group comparisons revealed a continuous decline in all domain scores in the intervention group at each successive time point from T1 to T3 (all *P* < 0.05) (see Table 7).

**TABLE 7:** Comparison of health-related quality of life between two groups (score, mean ± SD).

Time	Group	Symptom domain	Activity domain	Impact domain	Total score
T0	Control (*n* = 150)	62.5 ± 14.2	70.3 ± 15.0	55.8 ± 13.6	62.9 ± 12.3
	Intervention (*n* = 150)	63.1 ± 13.9	71.0 ± 14.7	56.2 ± 13.2	63.5 ± 11.9
	/	*t* = 0.34	*t* = 0.41	*t* = 0.24	*t* = 0.39
	/	*P* = 0.734	*P* = 0.681	*P* = 0.809	*P* = 0.698
T1	Control (*n* = 150)	61.7 ± 13.8	69.5 ± 14.8	55.0 ± 13.2	62.0 ± 12.1
	Intervention (*n* = 150)	55.3 ± 13.1^a^	63.2 ± 14.0^a^	49.1 ± 12.8^a^	55.8 ± 11.6^a^
	Adjusted difference (95% CI)	–6.2 (–8.9, -3.5)	–6.1 (–9.0, -3.2)	–5.7 (-8.3, –3.1)	-6.0 (–8.4, –3.6)
	*P*	< 0.001	<0.001	< 0.001	<0.001
T2	Control (*n* = 150)	60.5 ± 13.6	68.8 ± 14.5	54.2 ± 13.0	61.1 ± 11.9
	Intervention (*n* = 150)	48.7 ± 12.7^ab^	56.3 ± 13.4^ab^	43.2 ± 12.0^ab^	49.4 ± 11.2^ab^
	Adjusted difference (95% CI)	–11.4 (–14.2, –8.6)	–12.0 (–14.9, –9.1)	-10.6 (–13.2, –8.0)	–11.3 (–13.8, –8.8)
	*P*	*P* < 0.001	*P* < 0.001	*P* < 0.001	*P* < 0.001
T3	Control (*n* = 150)	59.8 ± 13.4	68.1 ± 14.2	53.8 ± 12.7	60.3 ± 11.7
	Intervention (*n* = 150)	42.5 ± 12.0^abc^	49.2 ± 12.6^abc^	38.5 ± 11.5^abc^	43.4 ± 10.9^abc^
	Adjusted difference (95% CI)	–16.7 (–19.4, –14.0)	–18.2 (–21.0, –15.4)	–14.8 (–17.4, –12.2)	–16.3 (–18.8, –13.8)
	*P*	< 0.001	<0.001	< 0.001	<0.001

*^a^*Compared with the same group at T0 (*P* < 0.05);

*^b^*compared with the same group at T1 (*P* < 0.05);

*^c^*compared with the same group at T2 (*P* < 0.05). Adjusted difference = intervention group - control group, adjusted for age, sex, GOLD classification, smoking status, comorbidities, baseline CAT score, and baseline SGRQ score.

### 3.5 Knowledge level distribution of patients with COPD

**FIGURE 2:**
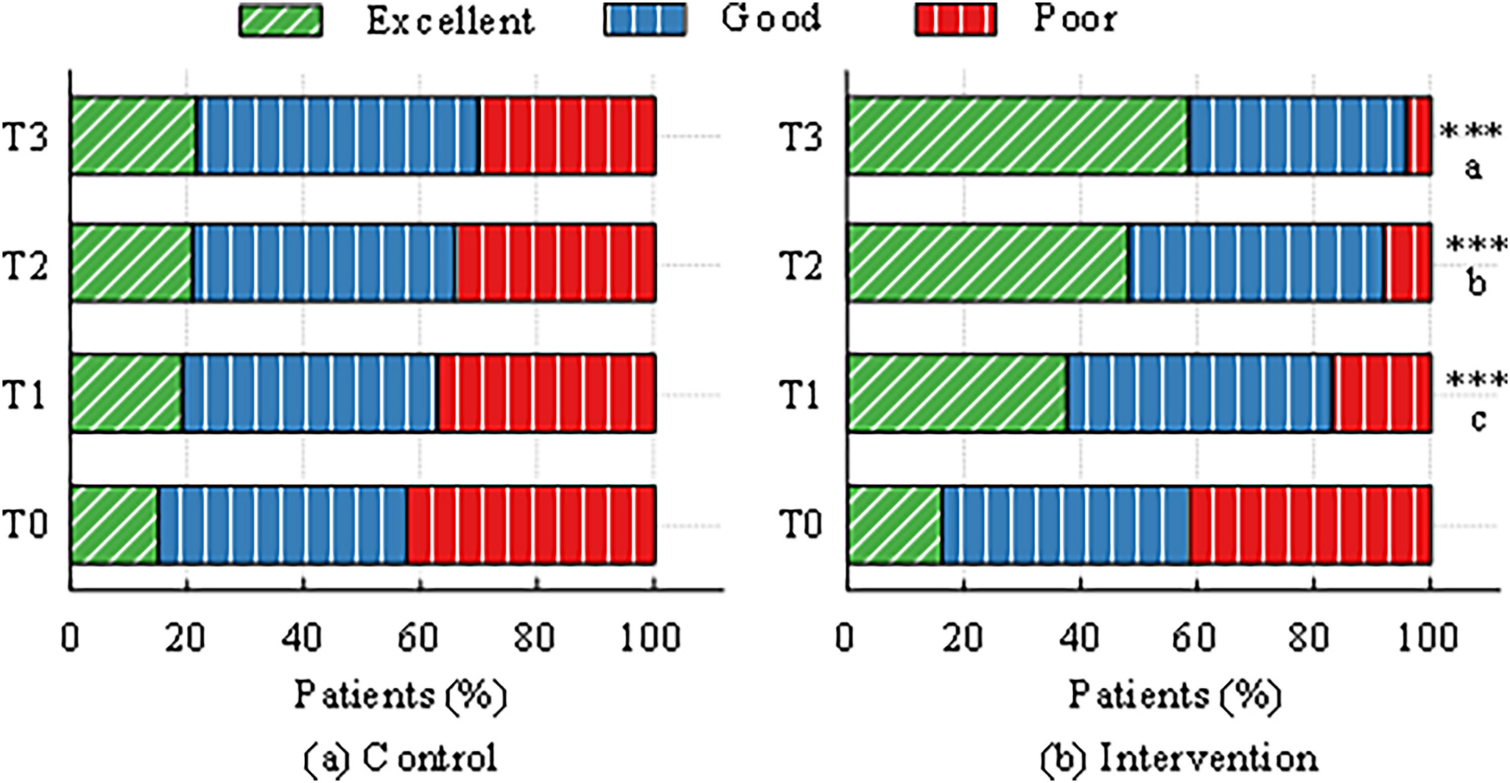
Distribution of COPD patient knowledge levels between the two groups. **(a)** Control group; **(b)** Intervention group. ***P < 0.001 between groups (intervention vs. control) at the same time point; a vs. T0, b vs. T1, c vs. T2 (all *P* < 0.05).

To further evaluate the impact of the intervention, the distribution of disease management knowledge levels was assessed in both groups across multiple time points. At T0, there was no statistical difference in knowledge score distribution between the groups at baseline (*P* > 0.05). Following the intervention, at T1, the proportion of participants with excellent knowledge in the intervention group increased substantially (36.7% vs. 16.7%), while the proportion of participants with poor knowledge decreased (16.6% vs. 41.6%, *P* < 0.001). At T2 and T3, the proportion of participants with excellent knowledge in the intervention group increased further, reaching 47.7 and 58.0%, respectively. Meanwhile, the proportion of participants with poor knowledge decreased to 9.3 and 5.3%, respectively, which was significantly lower than that observed in the control group (*P* < 0.001). Within-group analysis showed that the intervention group had a significant increase in the proportion of participants with excellent knowledge between T1 and T3 relative to baseline T0 (a, b, c, *P* < 0.05), whereas the proportion of participants with poor knowledge decreased steadily. No substantial changes in the distribution of knowledge levels in the control group (see Figure 2).

### 3.6 Medication adherence

At T0, there was no significant difference in the distribution of medication adherence based on the ARMS between the two groups (*P* = 0.914). At T1, a significant difference in adherence distribution emerged, with a significantly higher proportion of patients showing good adherence and a significantly lower proportion showing poor adherence in the intervention group compared with the control group (*P* < 0.001). At T2 and T3, these differences further widened, with a continuous increase in the proportion of patients with good adherence and a progressive decrease in poor adherence in the intervention group, both remaining significantly superior to those in the control group (all *P* < 0.001). In contrast, the adherence distribution in the control group showed no substantial change across the assessment time points (see Table 8).

**TABLE 8:** Distribution of medication adherence levels based on ARMS [n (%)].

Time point	Group	Good adherence	Moderate adherence	Poor adherence	χ ^2^	*P*-value
T0	Control (*n* = 150)	42 (28.0)	61 (40.7)	47 (31.3)	0.18	0.914
	Intervention (*n* = 150)	44 (29.3)	59 (39.3)	47 (31.4)		
T1	Control (*n* = 150)	46 (30.7)	58 (38.6)	46 (30.7)	18.62	< 0.001
	Intervention (*n* = 150)	72 (48.0)	58 (38.7)	20 (13.3)		
T2	Control (*n* = 150)	47 (31.3)	61 (40.7)	42 (28.0)	31.45	< 0.001
	Intervention (*n* = 150)	85 (56.7)	54 (36.0)	11 (7.3)		
T3	Control (*n* = 150)	49 (32.7)	62 (41.3)	39 (26.0)	48.97	< 0.001
	Intervention (*n* = 150)	96 (64.0)	50 (33.3)	4 (2.7)		

### 3.7 Self-care ability

There was no significant difference in baseline ESCA scores between the two groups (*P* = 0.612). From T1 onward, ESCA scores were significantly higher in the intervention group than in the control group (*P* < 0.001). After adjustment for covariates, the between-group difference at T1 was +11.5 points (95% CI: +9.7, +13.3). During follow-up, ESCA scores in the intervention group increased continuously, whereas no significant changes were observed across time points in the control group (all *P* > 0.05). By T3, the adjusted between-group difference had increased to +26.4 points (95% CI: +24.3, +28.5). These findings suggest that the benefits of the intervention accumulated over time and became progressively stronger (see Table 9).

**TABLE 9:** Self-care ability of patients in two groups (score, mean ± SD).

Time point	Control group (*n* = 150)	Intervention group (*n* = 150)	Adjusted between-group difference (95% CI)	*P*-value
T0	92.3 ± 8.5	91.7 ± 8.8	–	0.612
T1	93.6 ± 8.2	105.4 ± 9.1^a^	+11.5 (+9.7, +13.3)	< 0.001
T2	94.2 ± 8.0	114.8 ± 9.6^ab^	+20.1 (+18.2, +22.0)	< 0.001
T3	95.0 ± 8.1	122.1 ± 10.2^abc^	+26.4 (+24.3, +28.5)	< 0.001

*^a^*Compared with the same group at T0 (*P* < 0.05);

*^b^*compared with the same group at T1 (*P* < 0.05);

*^c^*compared with the same group at T2 (*P* < 0.05). Adjusted difference = intervention group - control group, adjusted for age, sex, GOLD classification, smoking status, comorbidities, baseline CAT score, and baseline SGRQ score.

## 4 Discussion

Individuals with COPD experience recurrent symptom burden and diminished quality of life, while also imposing prolonged caregiving demands (17). Previous studies indicated that single health education sessions or routine follow-up were insufficient to improve adherence and caregiver experience, suggesting the need for more systematic and continuous nursing models (18). The IKAP theory provides a structured pathway for chronic disease management. However, in the field of COPD, evidence-based studies focusing on both patient and caregiver outcomes remain limited (19). Based on this, the present study adopted a retrospective controlled design, dividing 300 patients into a routine care group and an IKAP-based continuous nursing group. Differences in symptom management, quality of life, adherence, self-care ability, and caregiver burden were compared. Overall results suggested that IKAP-based continuous nursing was associated with better outcomes than routine care in multiple outcomes, providing new evidence for integrated COPD management.

At the patient level, IKAP-based continuous nursing was associated with reductions in CAT and SGRQ scores, reflecting improvements in COPD-related health status. The intervention group showed a significant decline in CAT scores from T1 to T3 compared with the control group (all *P* < 0.001), showing a steady decline across time points. These findings suggested that systematic care may be associated with improvements in health status at an early stage and help sustain short-term benefits by reinforcing health behaviors and adherence. Mechanistically, the IKAP model enhanced disease awareness and self-management initiative through continuous education and attitude intervention. Research suggested that continuity care based on the IKAP theory was associated with increased adherence to exercise prescriptions (20). This finding suggested that systematic knowledge delivery, attitude reinforcement, and practice supervision may have contributed to greater persistence in daily rehabilitation training. Previous studies have demonstrated that exercise adherence is closely related to improvements in lung function, enhancement of exercise endurance, and reduction of symptom burden (21). Therefore, the differences in adherence observed in this study not only reflected the implementation strength of the nursing model but also provided a mechanistic explanation for better symptom management and improved quality of life. Outcomes related to health-related quality of life supported this result. The intervention group scored lower than the control group in symptom, activity, and impact domains. At T3, the total score decreased to 43.4 ± 10.9, while the control group remained at 60.3 ± 11.7. This difference indicated that continuous nursing improved not only respiratory symptoms but also physical activity and social participation. In contrast to previous studies, traditional education was often limited to knowledge transfer and rarely achieved multidimensional improvements (22). In contrast, the present study emphasized both attitude and practice, forming a positive cycle from cognition to behavior and addressing prior gaps. Moreover, enhanced medication adherence and self-care ability were also important contributions. These findings suggested that continuous nursing improved patient self-management through individualized medication guidance and behavior supervision. Unlike previous studies that relied only on medication reminders, this study highlighted the advantage of IKAP theory in promoting overall adherence and long-term self-care ability (23, 24). From a clinical perspective, the minimum clinically important difference for the CAT score is generally accepted as 2 points, and approximately 4 points for the SGRQ total score. In the present study, the between-group differences in both CAT and SGRQ total scores at T3 exceeded these respective thresholds, suggesting that the observed improvements were not only statistically significant but also clinically meaningful.

At the caregiver level, the study demonstrated improvements in family burden through structured caregiver training and continuous family engagement. Caregiver burden, assessed by ZBI-22, declined progressively between T1 and T3 in the intervention group, reaching values at 6 months that were well below those of the control group (*P* < 0.001). Only slight improvement was observed in the control group. This suggested that knowledge training, skill guidance, and psychological support were associated with caregivers manage daily care tasks with less stress, and may have contributed to reducing both physical and psychological burden. Mechanistically, the “information” and “knowledge” components of the IKAP model not only improved patients’ disease understanding but also enhanced family support ability through caregiver training (completion rate 85.1% vs. 10.7%). This reduced anxiety and helplessness caused by lack of knowledge. In terms of knowledge acquisition, the proportion of caregivers at the “excellent” level in the intervention group rose from less than 20% at T0 to 58.0% at T3, while the “poor” level decreased to 5.3%. The control group maintained nearly 40% in the “poor” category. Unlike earlier studies that mostly targeted patients alone, this research emphasized family involvement, addressing the limitation of restricted intervention coverage (25). Dynamic push notifications and quizzes not only ensured completion rates but also promoted the practical application of knowledge, forming a pathway of “cognition–attitude–practice.” Notably, improvement in caregiver burden interacted positively with patient symptom control and quality of life. Stronger family support encouraged patients to adhere to exercise and medication regimens, while better patient health further reduced caregiver workload. This bidirectional mechanism was fully validated in the study and provided evidence for patient–family collaborative interventions. Compared with prior research focusing on single outcomes, the innovation of this study lay in the dual emphasis on patients and caregivers, with the IKAP framework achieving comprehensive improvement (26).

In summary, IKAP-based continuous nursing was associated with improvements in symptom management, quality of life, self-care ability, and medication adherence in COPD patients, as well as reductions in caregiver burden and enhancements in disease knowledge. The mechanism relied on full-chain management through information collection, knowledge transfer, attitude reinforcement, and practice implementation, empowering both patients and caregivers. The strength of the study was the inclusion of patients and caregivers as a whole intervention unit, with multidimensional outcomes confirming the practical value of the IKAP framework. Its contribution was the provision of a structured and feasible nursing pathway that overcame the limitations of traditional point-based education. Several limitations should be acknowledged. First, the retrospective and non-randomized design introduced the possibility of selection bias. Systematic differences may have existed between groups in unmeasured factors, such as willingness to engage in self-management and the level of family caregiver involvement. Although baseline characteristics were comparable, residual confounding could not be completely excluded. Therefore, the findings should be interpreted as associations rather than causal relationships. Second, outcome data were mainly obtained from electronic medical records and self-reported questionnaires collected through the nursing management platform, which may have introduced information bias. Third, the follow-up period was limited to 6 months, and the long-term sustainability of the intervention effects remains unclear. Finally, as this was a single-center study, the generalizability of the results was limited. Future multicenter prospective randomized controlled studies are needed to further validate these findings. Overall, the study provided supporting evidence for the importance of continuous nursing in COPD management, provided new insights for clinical practice, and laid the foundation for future multicenter and prospective validation.

## Data Availability

The raw data supporting the conclusions of this article will be made available by the authors, without undue reservation.
